# Clinical Resistant Strains of *Enterococci* and Their Correlation to Reduced Susceptibility to Biocides: Phenotypic and Genotypic Analysis of Macrolides, Lincosamides, and Streptogramins

**DOI:** 10.3390/antibiotics12030461

**Published:** 2023-02-24

**Authors:** Amr Selim Abu Lila, Tareq Nafea Alharby, Jowaher Alanazi, Muteb Alanazi, Marwa H. Abdallah, Syed Mohd Danish Rizvi, Afrasim Moin, El-Sayed Khafagy, Shams Tabrez, Abdullah Ali Al Balushi, Wael A. H. Hegazy

**Affiliations:** 1 Department of Pharmaceutics, College of Pharmacy, University of Ha’il, Ha’il 81442, Saudi Arabia; 2 Molecular Diagnostics and Personalized Therapeutics Unit, University of Ha’il, Ha’il 81442, Saudi Arabia; 3 Department of Pharmaceutics and Industrial Pharmacy, Faculty of Pharmacy, Zagazig University, Zagazig 44519, Egypt; 4 Department of Clinical Pharmacy, College of Pharmacy, University of Ha’il, Ha’il 81442, Saudi Arabia; 5 Department of Pharmacology and Toxicology, College of Pharmacy, University of Ha’il, Ha’il 81442, Saudi Arabia; 6 Department of Pharmaceutics, College of Pharmacy, Prince Sattam bin Abdulaziz University, Al-Kharj 11942, Saudi Arabia; 7 Department of Pharmaceutics and Industrial Pharmacy, Faculty of Pharmacy, Suez Canal University, Ismailia 41522, Egypt; 8 King Fahd Medical Research Center, King Abdulaziz University, Jeddah 21589, Saudi Arabia; 9 Department of Medical Laboratory Sciences, Faculty of Applied Medical Sciences, King Abdulaziz University, Jeddah 21589, Saudi Arabia; 10 Pharmacy Program, Department of Pharmaceutics, Oman College of Health Sciences, Muscat 113, Oman; 11 Department of Microbiology and Immunology, Faculty of Pharmacy, Zagazig University, Zagazig 44519, Egypt; 12 Pharmacy Program, Department of Pharmaceutical Sciences, Oman College of Health Sciences, Muscat 113, Oman

**Keywords:** *Enterococci*, macrolides, lincosamides, streptogramins, *Enterococci faecalis*, *Enterococci faecium*, MLS phenotypes, genotyping, biocides

## Abstract

*Enterococci* are troublesome nosocomial, opportunistic Gram-positive cocci bacteria showing enhanced resistance to many commonly used antibiotics. This study aims to investigate the prevalence and genetic basis of antibiotic resistance to macrolides, lincosamides, and streptogramins (MLS) in *Enterococci*, as well as the correlation between MLS resistance and biocide resistance. From 913 clinical isolates collected from King Khalid Hospital, Hail, Saudi Arabia, 131 isolates were identified as *Enterococci* spp. The susceptibility of the clinical enterococcal isolates to several MLS antibiotics was determined, and the resistance phenotype was detected by the triple disk method. The MLS-involved resistance genes were screened in the resistant isolates. The current results showed high resistance rates to MLS antibiotics, and the constitutive resistance to all MLS (cMLS) was the most prevalent phenotype, observed in 76.8% of resistant isolates. By screening the MLS resistance-encoding genes in the resistant isolates, the erythromycin ribosome methylase (*erm*) genes that are responsible for methylation of bacterial 23S rRNA were the most detected genes, in particular, *ermB*. The *ereA* esterase-encoding gene was the most detected MLS modifying-encoding genes, more than *lnuA* (adenylation) and *mphC* (phosphorylation). The minimum inhibitory concentrations (MICs) of commonly used biocides were detected in resistant isolates and correlated with the MICs of MLS antibiotics. The present findings showed a significant correlation between MLS resistance and reduced susceptibility to biocides. In compliance with the high incidence of the efflux-encoding genes, especially *mefA* and *mefE* genes in the tolerant isolates with higher MICs to both MLS antibiotics and biocides, the efflux of resistant isolates was quantified, and there was a significant increase in the efflux of resistant isolates with higher MICs as compared to those with lower MICs. This could explain the crucial role of efflux in developing cross-resistance to both MLS antibiotics and biocides.

## 1. Introduction

*Enterococci* are facultatively anaerobic Gram-positive opportunistic bacteria that are normally found in the human gastrointestinal tract and the female genital tract and abundant in the environment, such as in soil and water [1]. According to Lancefield classification, Enterococci were classified as group D Streptococci based on the carbohydrate substances in their cell walls [2]. *E. faecalis* and *E. faecium* are the most important Enterococcal species and are among the foremost causes of nosocomial infections, causing severe infections such as septicemia and endocarditis [3,4]. The unusual adaptation conferred the survival and persistence of *Enterococci* in adverse environments as inanimate surfaces in hospitals and at sites of infections [3,5,6]. This survival ability allows *Enterococci* to interact with other overtly resistant bacteria acquiring additional resistances on mobile elements. Noticeably, a quarter of a genome of additional DNA obtained by mobile elements certainly allows Enterococci to persist and spread in the hospital setting and resist antimicrobials causing hostile infections [5,7,8,9,10]. The swift increase in the resistance among hospital-adapted enterococci to a wide diversity of antimicrobials has rendered nosocomial infections a leading therapeutic challenge [1,11,12,13].

Macrolide and lincosamide antibiotics are chemically distinct antibiotic groups but have similar modes of action. For years, these antibiotics represented an alternative to penicillin and cephalosporins; however, the development of macrolide resistance limited the use of these antibiotics to certain indications [14,15,16,17]. Naturally occurring macrolides comprise two amino or neutral sugars attached to a 14–16 membered lactone ring. Newer semisynthetic macrolides had substitutions on the lactone ring that improved acid stability and antimicrobial activity [18]. Lincosamides include the naturally occurring lincomycin and its semi-synthetic derivative, clindamycin. Although lincosamides lack the lactone ring of macrolides, lincosamides share the same mechanism of action as macrolides in targeting 50S bacterial sub-ribosomal unit [14]. Macrolides and lincosamides inhibit bacterial protein synthesis by reversibly binding to the 50S subunit of the bacterial ribosome, [14] stimulating the dissociation of the peptidyl-tRNA from the ribosomes during elongation, causing chain termination [18]. Another antibiotics class that reversibly binds to the 50S bacterial ribosomal subunit is streptogramins [19]. Streptogramin antibiotics act by inhibiting bacterial protein synthesis and are divided into two groups, streptogramin A and streptogramin B, which work together synergistically to produce a bactericidal effect [19,20]. Streptogramins are synthesized by different *Streptomyces* spp., where group A streptogramins contain 23-membered unsaturated rings with lactone and peptide bonds, and group B streptogramins are cyclic hexa- or hepta-depsipeptides produced [20].

Macrolide/lincosamide/streptogramin (MLS) resistance is increasing among the clinical isolates of Gram-positive bacteria, and the multiplicity of resistance mechanisms of these drugs results in a variety of resistance phenotypes [14]. Three different mechanisms of the acquired MLS resistance have been found in Gram-positive bacteria: (1) target-site modification by methylation or mutation of 23S rRNA, (2) efflux of the antibiotic, and (3) drug inactivation. The most clinically important and widespread resistance mechanisms are the methylation of the 23S rRNA ribosomal subunit and the drug efflux [14,18,21]. While modifications confer broad-spectrum resistance to macrolides and lincosamides, enzymatic modification affects only structurally related antibiotics [14,21].

The improper use, either suboptimal or misuse, of antibiotics in human and veterinary medicine is considered the major cause of antibiotic resistance [22,23]. Recently, the use of biocides in many products as household products, plastics, cosmetics, etc., has been reported as a risk factor contributing to antimicrobial resistance development in humans and the environment [24]. Biocidal agents used for disinfection are usually not assumed to enhance the cross-resistance to antibiotics, although resistant or more tolerant bacteria were isolated from in-vitro cultures after exposure to suboptimal or sublethal levels of biocides [25]. The present study aimed to determine the most prevalent resistance patterns, phenotypes, and the most predominant resistance genes to MLS antibiotics among the collected clinical *Enterococci* isolates. Moreover, it is aimed to recognize the correlation between the resistance to MLS and the susceptibility to frequently used biocides.

## 2. Results

### 2.1. Isolation and Identification of Enterococci *spp.*

**Figure 1:**
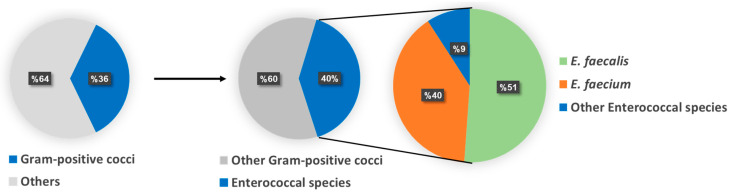
Incidence of Enterococcal spp. Among 913 clinical samples, 35.6% were Gram-positive cocci that contained about 40.3% *Enterococci* spp. Further, the Enterococcal spp. were presumptively distinguished into *E. faecalis* (51.1%), *E. faecium* (39.7%), or other *Enterococci* species (9.2%).

Three hundred and twenty-five (35.6%) Gram-positive cocci isolates were recovered from 913 clinical samples. One hundred and thirty-one from isolated Gram-positive cocci (40.3%) showed darkening of the medium around the bacterial colonies, indicating *Enterococcus* spp., and further biochemical identifications were conducted [26,27]. The Enterococcal spp. isolates that did not ferment arabinose and showed growth in 0.04% tellurite were considered *E. faecalis.* In contrast, the isolates that did not grow in 0.04% tellurite and ferment arabinose were considered *E. faecium*. The Enterococcal spp. isolates that showed darkening on bile esculin agar and showed variable results for other tests listed in Table 1 were considered other *Enterococci* spp. Among 131 *Enterococci* isolates, 67 (51.1%), 52 (39.7%), and 12 (9.2%) were presumptively identified as *E. faecalis*, *E. faecium*, or other *Enterococci* species, respectively (Figure 1).

### 2.2. Susceptibility to MLS

**Figure 2:**
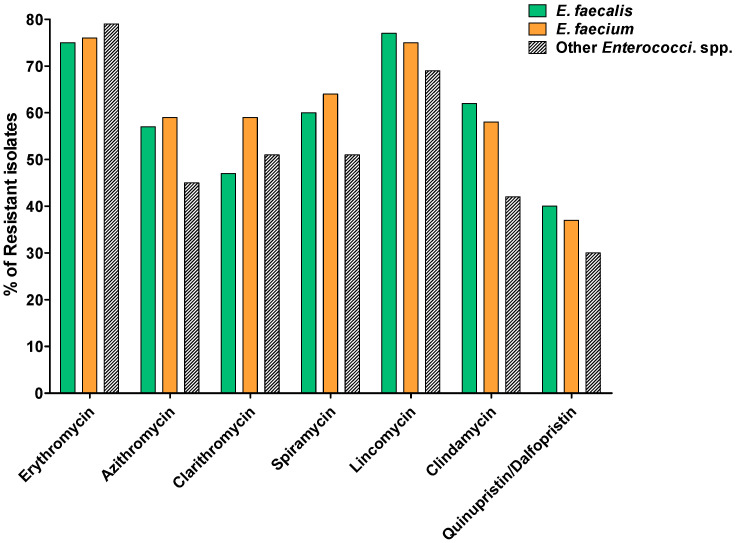
Percentages of resistance to tested MLS antibiotics. The chi-square test was used to compare the difference in the percentages of resistant isolates to tested antibiotics. There was no significant difference in the resistance of different *Enterococci* spp. to tested antibiotics; ($\chi^{2}{}_{\left(10\right)}$ = 4.98, *p* = 0.892).

**Figure 3:**
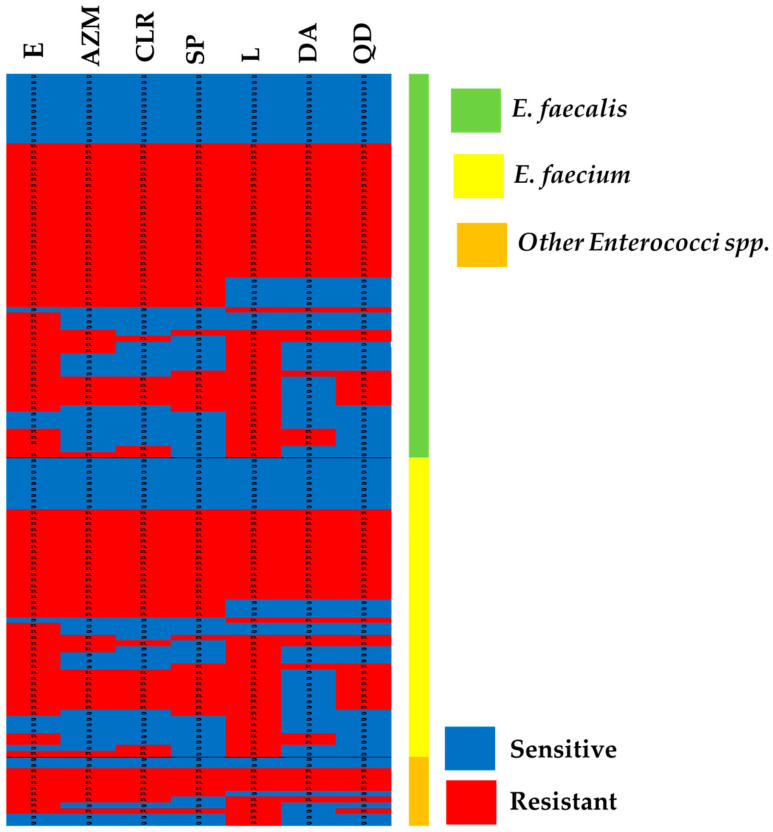
The resistance patterns to the tested MLS antibiotics. The heat map represents the resistance, where red represents the resistant isolates, and blue represents the sensitive isolates. The highest resistance was observed to erythromycin and lincomycin in both *E. faecalis* and *E. faecium.* E = Erythromycin, AZM = Azithromycin, CLR = Clarithromycin, SP = Spiramycin, L = Lincomycin, DA = Clindamycin, and QD = Quinupristin/Dalfopristin.

The Enterococcal isolates were tested for their susceptibility to erythromycin, azithromycin, clarithromycin, spiramycin, lincomycin, clindamycin, and quinupristin/dalfopristin by disk diffusion method. Chi-square ($\chi^{2}{}_{\left(12\right)}$ = 6.42, *p* = 0.89) is not statistically significant, indicating no significant difference in the resistance of different *Enterococci* spp. to tested antibiotics (Figure 2). The higher resistance values were observed for erythromycin and lincomycin (about 76%). Furthermore, *E. faecalis* and *E. faecium* were more resistant than other *Enterococci* spp. The detailed patterns of resistance to the MLS antibiotics are provided in Table S1 and shown in Figure 3. Importantly, the resistance to all tested MLS antibiotics was observed in 43 (32.8%) isolates, while 22 (16.8%) isolates were sensitive to all antibiotics. The higher resistance rates were observed in all the tested macrolides; it was observed in 66 (50.4%) isolates. The resistance rates to streptogramins and lincosamides were 48% and 43.5%, respectively.

### 2.3. MLS Resistance Phenotypes

One hundred and eight *Enterococci* isolates that showed resistance to macrolides, lincosamides, and/or streptogramins were selected for further investigation of the resistance phenotypes and genotypes. These isolates comprised 55 *E. faecalis*, 43 *E. faecium*, and 10 other *Enterococci* spp.

**Figure 4:**
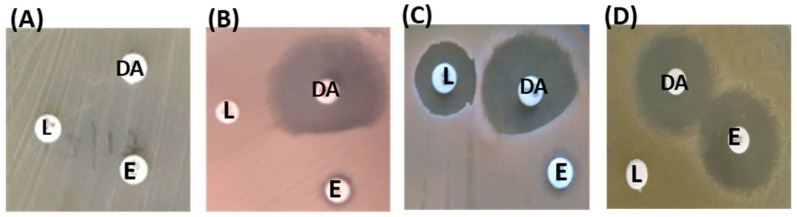
MLS resistance phenotypes. The inhibition zones to erythromycin (E), clindamycin (DA), and lincomycin (L) were observed. (**A**) Constitutive macrolide/lincosamide/streptogramin resistance (cMLS) phenotype: isolates resistant to the three drugs. (**B**) Inducible macrolide/lincosamide/streptogramin resistance (iMLS) phenotype: isolates show flattening or blunting of the shape of the clindamycin zone and are resistant to erythromycin and lincomycin. (**C**) M phenotypes: isolates resistant to erythromycin only but sensitive to clindamycin and lincomycin. (**D**) L phenotype: isolates resistant to lincomycin and sensitive to clindamycin and erythromycin. E: erythromycin, DA: clindamycin, and L: lincomycin.

The inhibition zones between erythromycin, clindamycin, and lincomycin disks were measured in mm, and the triple disk diffusion method was employed to determine the resistance phenotype of the resistant isolates. The ingrowth within zones up to the edges of each erythromycin, clindamycin, and lincomycin disk was considered constitutive macrolide/lincosamide/streptogramin resistance (cMLS) phenotype. Flattening or blunting of the shape of the clindamycin zone indicates inducible macrolide/lincosamide/streptogramin resistance (iMLS) phenotype. Isolates resistant to erythromycin only but sensitive to clindamycin and lincomycin were considered to belong to M phenotypes. Resistance to lincomycin with sensitivity to clindamycin and erythromycin was considered an L phenotype (Figure 4). Out of the 108 selected isolates, 83 (76.8%), 19 (17.6%), 4 (3.7%), and 2 (1.9%) isolates showed cMLS, M, iMLS, and L resistance phenotypes, respectively, as shown in Table 2.

### 2.4. MLS Resistance Genotypes

Resistant bacteria employ several mechanisms to resist MLS antibiotics, including (i) changing the antibiotic’s bacterial target by methylation of 23S rRNA, (ii) efflux, and (iii) production of antibiotic’s modifying enzymes as esterase, adenylating, and phosphorylating enzymes [14,28]. In this context, the PCR was used to detect the *erm* genes (*ermA*, *ermB*, and *ermC*), which are responsible for methylating the 23S rRNA protecting bacteria from MLS antibiotics. The genes *msrA*, *mefA*, and *mefE* are efflux-encoding genes and are responsible for pumping out MLS antibiotics. Furthermore, the genes encode the enzymes that hydrolyze (*ereA*), adenylate (*lnuA*), or phosphorylate (*mphC*) MLS were detected.

**Figure 5:**
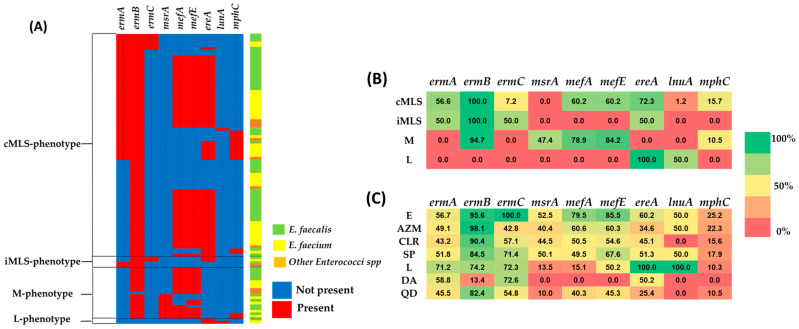
MLS resistance genotypes. (**A**) The distribution of the genes involved in the resistance to MLS in the different resistant isolates. The *erm* genes, particularly *ermB*, were the most predominant in all resistant isolates. (**B**) Heat map represents the percentage of the MLS resistance genes in different phenotypes. (**C**) Heat map represents the distribution of resistance genes in the resistance to different antibiotics.

The current finding revealed the detection of all the tested genes in the resistant isolates, as shown in Figure 5A and detailed in Table S2. The most detected genetic base of resistance was the methylation of 23S rRNA, as the *erm* genes were the most abundant detected genes in 97.2% resistant isolates. The most detected *erm* gene is *ermB* which was detected in 97.2% of resistant isolates, followed by *ermA* and *ermC*, which were found in 44.5% and 7.5% of resistant isolates. Interestingly, the coexistence of *ermA*, *ermB*, and *ermC* genes was observed only in 5.6%, which all showed cMLS phenotype, while the coexistence of *ermA* and *ermB* was observed in 44.5% (43.5% cMLS- and 1% iMLS-phenotypes) of resistant isolates. On the other hand, the coexistence of *ermB* and *ermC* was observed in 7.5% (5.6% cMLS- and 1.9% iMLS-phenotypes). It is worth mentioning that the only *erm* gene detected in M-phenotype isolates was the *ermB* gene, while no *erm* genes were detected in L-phenotype isolates. Meanwhile, the genes responsible for the breakdown or efflux of MLS were detected at 67.6% or 66.7%, respectively. The esterase, adenylation, and phosphorylation encoding genes *ereA*, *lnuA*, or *mphC* were found in 59.3%, 1.9%, or 13.9% of the resistant isolates, respectively. The efflux encoding genes *msrA*, *mefA*, or *mefE* were detected in 8.4%, 60.2%, or 61.1% of resistant isolates, respectively.

Furthermore, the prevalence of the resistant genes in different resistant phenotypes was screened (Figure 5B). The *ermB* was observed in 100% of cMLS-, iMLS-, and M-phenotypes and was absent in L-phenotype isolates. On the other hand, *lunA* and *ereA* genes were only observed in L-phenotype isolates. While the *erm* genes and *ereA* gene were the most detected in cMLS- and iMLS-phenotypes, the efflux genes and only the *ermB* gene were predominant in M-phenotypes. In L-phenotypes, *ereA* and *lnuA* were the only detected genes, 100% and 50%, respectively.

Additionally, the resistance-encoding genes were screened in the resistant strains of each MLS antibiotic (Figure 5C). The *ermB* was the highest detected gene in the resistant strains to tested macrolides, lincomycin, and streptogramin. The *lnuA* gene was detected in the two strains resistant to lincomycin; one was cMLS-phenotype, and the other was L-phenotype. In the clindamycin-resistant strains, the only detected genes were *erm* genes and *ereA* genes. The efflux genes were observed mainly in the macrolide- and lincomycin-resistant strains. The phosphorylation (*mphC*) was less detected in contrast to the hydrolysis of lactone ring (*ereA*) as a mechanism to break down the MLS antibiotics.

### 2.5. The Minimum Inhibitory Concentrations (MICs) of MLS and Biocides

The MICs (µg/mL) of the tested antibiotics were determined by the agar dilution method. The MIC ranges, MIC_50_ and MIC_90,_ are presented in Table S3. It is observed that the lowest MIC that is required, 50% or 90%, inhibits bacterial growth observed with clarithromycin, spiramycin, and quinupristin/dalfopristin. The MICs ranges were 0.125–1024 µg/mL for all the tested MLS antibiotics. Furthermore, the MICs of the resistant isolates were detected against triclosan, cetrimide, glutaraldehyde, thiomersal, chlorocresol, and povidone-iodine, which represent different biocides (Table S4). The MICs ranges of triclosan, cetrimide, glutaraldehyde, thiomersal, chlorocresol, and povidone-iodine to resistant isolates were 0.1–0.7 µg/mL, 0.5–10 µg/mL, 0.1–1.8 µg/mL, 0.2–7 µg/mL, 150–600 µg/mL, and 900–5600 µg/mL, respectively.

#### The Correlation between MLS Resistance and Reduced Susceptibility to Biocides

**Figure 6:**
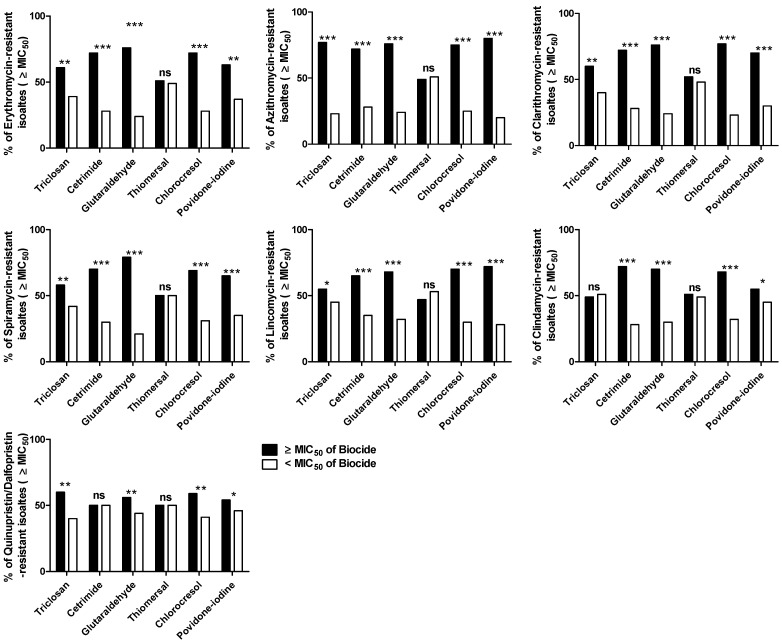
The percentages of reduced susceptible isolates to biocides (MIC ≥ MIC_50_) in the antibiotics-resistant isolates with MIC ≥ MIC_50_. The chi-square test was used to compare the difference in the percentage of antibiotic-resistant isolates with MIC above or below the MIC_50_ of tested biocides. It was observed that in the antibiotic-resistant isolates, the percentages of reduced susceptible isolates to all biocides except thiomersal were increased significantly. ns: *p* > 0.05, *: *p* ≤ 0.05, **: *p* ≤ 0.01, ***: *p* ≤ 0.001.

Enterococcal isolates were categorized as reduced susceptible or susceptible to the MLS antibiotics or biocides relative to the biocides MIC_50_ [29]. The reduced susceptibility was considered for isolates that were inhibited by antibiotics or biocides at concentrations above MIC_50_. There were 38 isolates that showed higher MIC ≥ MIC_50_ to all the tested antibiotics and also to all biocides. To correlate between the resistance to antibiotics and the reduced susceptibility to biocides for the isolates, the percentage of antibiotic-resistant isolates among biocides susceptible (with MIC below MIC_50_) and biocides tolerant (with MIC above MIC_50_) isolates were compared in the isolates that showed MIC ≥ MIC_50_ of antibiotics. The chi-square test was used to compare the difference in the percentage of antibiotic-resistant isolates with MIC above or below the MIC_50_ of tested biocides. Chi-square values were statistically significant in most antibiotic-resistant isolates, indicating a significant difference between biocide tolerant (MIC above MIC_50_) and susceptible (MIC below MIC_50_) isolates. In other words, isolates that were inhibited by antibiotics at higher MIC ≥ MIC_50_ were significantly inhibited by higher concentrations of biocides MIC ≥ MIC_50_ (Figure 6). It is worth mentioning that there was no significant correlation between the reduced susceptibility to thiomersal and the resistance development to all tested MLS antibiotics, as there was no significant difference between the numbers of isolates that showed MIC to thiomersal <MIC_50_ and >MIC_50_ in all MLS resistant isolates.

**Figure 7:**
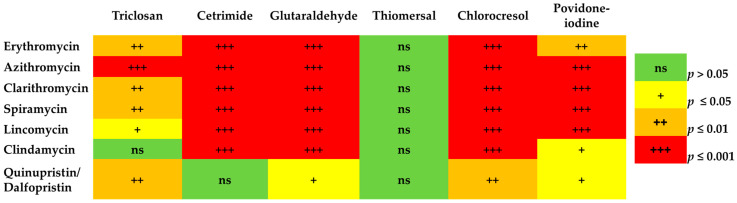
The Correlation between the reduced susceptibility to biocides and antibiotic resistance was calculated. Pearson’s correlation coefficients of pairwise comparison were employed to assess the correlation between MIC values for antibiotics and biocides of individual isolates, which showed MIC ≥ MIC_50_. There were significant correlations between the numbers of resistant isolates to antibiotics and the number of isolates with increased susceptibility to all biocides except thiomersal.

In addition, Pearson’s correlation coefficients between MIC values for MLS antibiotics and biocides of individual isolates. There was a stronger correlation between increasing MIC values for antibiotics and biocides (*p* < 0.05 was considered significant) (Figure 7). Significantly, there were correlations between reduced susceptibility to cetrimide, glutaraldehyde, chlorocresol, and povidone-iodine and resistance to all tested antibiotics. The reduced susceptibilities to triclosan and cetrimide were significantly correlated to all tested antibiotics except clindamycin, and quinupristin/dalfopristin, respectively. Furthermore, there was no significant correlation between reduced susceptibility to thiomersal and resistance to all tested antibiotics in all resistant isolates.

### 2.6. The Distribution of Resistant MLS Genes in the Resistant Isolates with MIC ≥ MIC_50_

**Figure 8:**
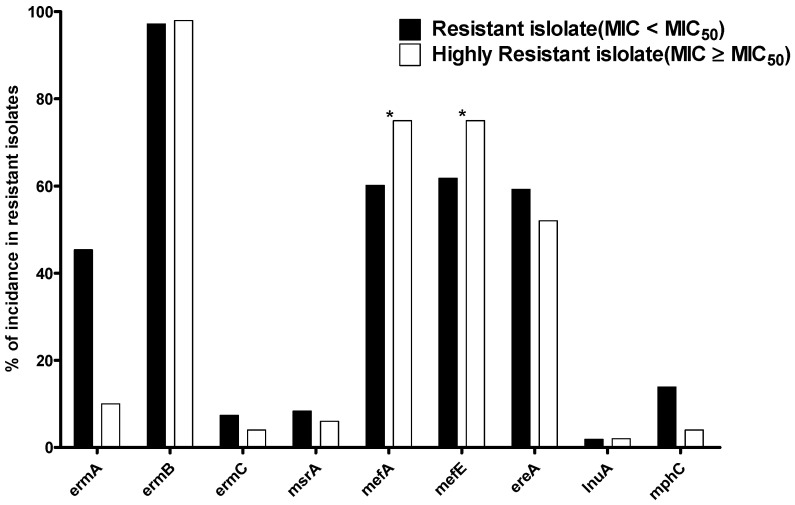
The distribution of MLS genes in the highly resistant isolates that showed MIC ≥ MIC_50_. The efflux encoding genes *mefA* and *mefE* were significantly increased in the highly resistant isolates (MIC ≥ MIC_50_) than in other resistant isolates with MIC < MIC_50_. That could explain the increased role of efflux in resistance to both antibiotics and biocides. * *p* < 0.05.

In order to explore the most involved resistance mechanism in the resistance to both MLS antibiotics and biocides, the distribution of MLS genes was screened in the antibiotic-resistant isolates with MIC ≥ MIC_50_ and, at the same time, showed reduced susceptibility to biocides with MIC ≥ MIC_50_. The genes involved in the three resistance mechanisms were found in the highly resistant isolates. However, *ermB*, *mefA*, *mefE*, and *ereA* genes were the most detected genes. Chi-square test was employed to statistically compare the incidence of resistant genes in the highly resistant isolates (MIC ≥ MIC_50_) and their incidence in the rest of the resistant isolates. Considering that *ermB* was the most detected gene in all resistant isolates, no significant difference existed between its incidence in the resistant and highly resistant isolates with MIC ≥ MIC_50_. Only the efflux encoding genes *mefA* and *mefE* were significantly increased in the highly resistant isolates that showed higher MIC ≥ MIC_50_, which could indicate that the increased resistance is owed mainly to the enhancement of the bacterial efflux to both MLS and biocides (Figure 8).

### 2.7. Efflux Assay in MLS Resistant Isolates

**Figure 9:**
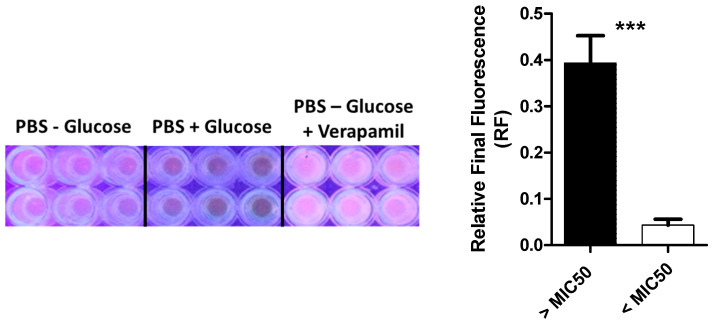
Increased efflux in highly resistant isolates. A quantitative fluorometric assay of EtBr efflux was performed for 20 high-resistant isolates (MIC > MIC_50_ to MLS antibiotics and biocides) against 20 resistant isolates (MIC < MIC_50_ to MLS antibiotics and biocides). The efflux assay was performed at conditions that cause maximum accumulation of EtBr in the presence of efflux pump inhibitor verapamil and limited energy supply (absence of glucose and low temperature). The efflux of EtBr is presented in terms of relative fluorescence (RF), which is obtained from the comparison between the fluorescence observed for the bacteria in the presence or absence of glucose and the control in which the cells are exposed to conditions of minimum efflux in the absence of glucose and presence of verapamil. All fluorescence readings were made at excitation and emission wavelengths for EtBr (530 nm and 585 nm, respectively). All data were acquired in cycles of 60 s, during 1 h time intervals, and at 25 °C. Each experiment was conducted in triplicate, and the results obtained were averaged. The relative fluorescence was calculated for each isolate with MICs to biocides >MIC_50_ or <MIC_50_, and results were expressed as means ± standard deviation. *** *p*-value < 0.001 was considered significant using Student’s *t*-test. Significantly, the efflux of EtBr was increased in the isolates with MIC > MIC_50_, indicating high efflux activity that could explain high resistance to both biocides and antibiotics.

One of the mechanisms that confer cross-resistance of bacteria to both MLS antibiotics and biocides is the efflux mechanism. To evaluate the efflux efficiency, a quantitative assay of ethidium bromide (EtBr) efflux was performed for selected 20 high-resistant isolates (MIC > MIC_50_ for both antibiotics and biocides) against 20 resistant isolates with MIC < MIC_50_ for both antibiotics and biocides. The minimum concentration of EtBr producing maximum fluorescence ranged from 0.25–2 µg/mL. The quantitative fluorometric efflux assay of EtBr was performed for each isolate three repeats in the absence or presence of glucose and verapamil at concentrations 450–750 µg/mL. The results were expressed as relative fluorescence by comparing the fluorescence observed for the bacteria in the presence or absence of glucose and the control in which the cells are exposed to conditions of minimum efflux in the absence of glucose and the presence of verapamil. Each assay was performed in triplicate, and relative fluorescence data are presented as the means ± standard deviation. The relative fluorescence (RF) values of the isolates with MICs to biocides >MIC_50_ were significantly increased than the isolates with MICs < MIC_50_ (*p* < 0.0001), indicating the high efflux activities in the isolates which were highly resistant to both antibiotics and biocides (Figure 9).

## 3. Discussion

The current study aimed to determine the susceptibility of the local *Enterococci* clinical isolates to MLS antibiotics to determine the most prevalent resistance phenotypes and the most common genetic determinant of the resistance. About 40% of Gram-positive isolates were identified as *Enterococci* spp.; the majority were *E. faecalis* and *E. faecium* (51% and 40%, respectively). The antibiotic susceptibility testing revealed an increment of the resistance rates of the tested MLS, particularly macrolides, specifically erythromycin. Clindamycin is a chlorinated derivative of lincomycin, and it is one of the 20 most important antibiotics, which is abundantly prescribed for prophylaxis and treatment of anaerobic infections that could explain the development of resistance to it [30]. Generally, Gram-positive cocci, except *Enterococci*, are sensitive to lincomycin and clindamycin; however, increased plasmid-mediated Enterococcal resistance traits could be recognized in clinical isolates [30,31]. That agrees with our findings, which showed high resistance rates to lincomycin and clindamycin (about 78% and 60%, respectively). Enterococcal resistance to streptogramins has been observed worldwide [32,33,34,35], which complies with the present findings, which showed about 35% resistance in all tested *Enterococci* isolates.

Although MLS antibiotics are chemically distinct, they are usually considered together because most share overlapping binding sites on the 50S ribosomal subunit inhibiting the translation process. These antibiotics bind within the exit tunnel adjacent to the peptidyl transferase center and inhibit the progression of the nascent chain, making peptidyl-tRNA drop-off [36]. Even though many bacterial species acquire resistance genes that confer resistance to more than one MLS antibiotic [21], different antibiotics interact and bind with different rRNA residues, which may explain why a bacterium may be resistant to one MLS antibiotic but susceptible to another [37].

Three main mechanisms of acquired MLS antibiotics resistance have been described in Gram-positive bacteria. The first mechanism protects the bacterial ribosome from the drug binding by 23S rRNA methylation. It is a cross-resistance to all three structurally different MLS antibiotics owed to *erm* genes and can be expressed constitutively or inducible [21,38,39,40,41,42,43]. In the inducible resistance phenotype, bacteria produce inactive mRNA that becomes active only in the presence of a macrolide inducer [14,18,21]. The strains harboring an inducible erythromycin ribosome methylase (*erm*) genes are resistant to the inducers (14- and 15-membered ring macrolides) but remain susceptible to non-inducer macrolides (16-membered ring), lincosamides, and streptogramins B. In constitutive expression, active methylase mRNA is produced in the absence of an inducer, and the strains express cross-resistance to MLS antibiotics [14,18]. Resistance to macrolides and lincosamides can also be due to the mutations affecting 23S rRNA ribosomal proteins L4 and L22 [44]. Clinical isolates that are constitutively resistant to MLS antibiotics are widespread, particularly in methicillin-resistant strains [45]. Several studies monitored that the constitutive phenotype (cMLS) appears to be the most predominant type in Enterococcal-resistant isolates from patients [35,38,39,42,46]. The current findings revealed the prevalence of cMLS resistance phenotype (76.8%), followed by M-, iMLS-, and L-phenotypes (19.7%, 3.7%, and 1.9%, respectively).

Target-site modification takes place through the mutation or methylation of 23S rRN methyl transferase enzyme resulting in cross-resistance to MLS but not to oxazolidinones giving the MLS phenotype [47]. The MLS phenotype is exhibited by 33 different erm genes expressed constitutively or inducibly [18,21]. These genes are mostly borne on plasmids and transposons that are self-transferable. Four major classes of *erm* genes were detected in pathogenic bacteria: *ermA*, *ermB*, *ermC*, and *ermF* [18]. In this study, PCR screening for selected *erm* genes revealed the presence of all tested genes *ermB* (97.2%), *ermA* (44.5%), and *erm*C (7.5%). The *ermA* gene is commonly spread in methicillin-resistant isolates (MRSA) and is horizontally transferred by transposons [14], which is why its presence was documented in *Enterococci* [48,49,50,51]. The *ermB* expression is induced by macrolides, lincosamides, streptogramins [14,52], and even by ketolides [53,54]. This could explain the high frequency of *the ermB* gene among *Enterococci* isolates, taking into consideration that the majority of *ermB*-positive isolates displayed the cMLS phenotype [55,56]. Moreover, it has been demonstrated that *ermB* expression is induced by a wide range of MLS antibiotics [52], which agrees with the current data. The *ermB* gene was recognized in all the isolates that showed iMLS-phenotype. Conversely to the *ermB* gene expressed by a wide range of MLS antibiotics, *ermC* expression is induced by a few macrolides [57,58]. The *ermC* gene is mostly responsible for erythromycin resistance and is transferred by plasmids [14], which complies with the present findings, which showed *ermC* in all erythromycin-resistant isolates are mostly cMLS (5.6% cMLS- and 1.9% iMLS-phenotypes of all resistant isolates).

Gram-positive and -negative bacteria can resist diverse groups of antibiotics by producing drug-inactivating enzymes [59,60,61]. About 19 genes code esterase, lyases, transferases, and phosphorylases enzymes which modify and inactivate MLS antibiotics by hydrolyzing the lactone ring (*ere* genes), adenylating (*lnu* genes), acetylating (*vat* genes), or phosphorylating (*mph* genes) [21,62]. Unlike target modification, drug inactivation confers resistance to the structurally related antibiotics only [18], but none of the inactivating enzymes are unique to certain bacterial species [63]. Whereas esterase, phosphotransferases, acetyltransferases, hydrolases, and nucleotidyl transferases were identified in strains resistant to MLS antibiotics, these inactivating enzymes confer resistance to erythromycin and other 14- and 15-membered macrolides but not to lincosamides that represented as L phenotype [18].

The *ere* genes, especially the *ereA* gene, are the most distributed MLS-inactivating genes in both Gram-positive and -negative bacteria [21]. The current results revealed that the *ereA* gene had been detected in 59.3% of resistant isolates showing the cMLS-, iMLS-, and L-phenotypes isolates but not detected in M-phenotype isolates. The *mph*C gene has been detected in 13.9% of the resistant isolates that showed either the cMLS phenotype or M phenotype. Notably, the *lnu*A gene was only detected in 1.9% of isolates that showed cMLS- or L phenotype that can be possibly explained as the *lnu* gene involves phosphorylation and nucleotidylation of lincosamides resulting in high resistance to lincosamides but not macrolides [64]. Considering that the resistance mediated by *lnu*A and/or *lnu*B genes confer resistance to lincomycin but not clindamycin, it is expressed as L phenotype [14]; the *lnuA* gene was detected in all lincomycin-resistant isolates but not detected in any clindamycin-resistant isolate.

The efflux mechanism in which the bacteria pump out one or more MLS antibiotics is owed to about 17 efflux genes via either ATP-transporters or major facilitator transporters [21]. However, efflux pumps are compartments of the bacterial cell wall, and their responsible genes are located on the chromosomes; transferable elements are more often involved in the enhanced efflux of MLS [50,65,66]. Based on the amino acid sequence and source of energy, the bacterial efflux transporters are classified into five different superfamilies [13]. The active efflux of MLS antibiotics is responsible for partial cross-resistance to 14- and 15-membered macrolides and streptogramin B and is conferred most abundantly *msr*, *vga*, *mef*, *isa*, and other genes [21,67]. The efflux resistance is inducibly expressed by erythromycin and other 14- and 15-membered macrolides [14,21]. Clindamycin is neither an inducer nor a substrate for the pump; thus, the efflux genes carrying strains are fully susceptible [14]. The *mef* genes encode for efflux in macrolides and *msr* genes for efflux of macrolides and streptogramin B; they have been involved in the active efflux of MLS in Gram-positive cocci [65,68,69]. These genes may be located on the chromosomes but are more often associated with transferable elements [50,65,66]. Our results showed that *mefA*, *mefE*, and *msrA* were recognized in 60.2%, 61.1%, and 8.4%, respectively. Interestingly, all isolates showed M phenotype carried *msrA* or/and *mefA* and *mefE* genes. These results are in great accordance with other groups. Iannelli et al. and others showed that efflux pumps encoded by *mefA* and its allele *mefE* genes are among the most common mechanisms of resistance to macrolides (M phenotype) [14,28,69]. Furthermore, the *msr*A gene displays the inducible resistance to erythromycin, while macrolide efflux affected by *mef* genes was reported in Gram-positive cocci [50]. Efflux pumps responsible for macrolides resistance in *Enterococci* include *mefA* and *mefE* pumps, which are involved in the intrinsic resistance to lincosamides and streptogramins in *E. faecalis* [28].

In the current study, high resistance rates were not observed in MLS antibiotics but also different biocides. Cross-resistance to antibiotics and biocides can be conferred by induction of common resistance mechanisms [70], e.g., efflux pumps and transfer of resistance genes for antimicrobials and antibiotics on mobile genetic elements [24,71]. In this direction, it is intended to correlate the enhanced resistance to MLS antibiotics and biocides. The correlation between the resistance to antibiotics and the reduced susceptibility to biocides for the isolates was determined by comparing the percentage of antibiotic-resistant isolates among biocides less resistant (with MIC < MIC_50_) and biocide tolerant (with MIC ≥ MIC_50_) isolates [29]. Our results revealed a significant difference between biocide tolerant and biocide susceptible isolates in MLS resistant isolates. However, there is a significant statistical correlation between elevation in MICs to MLS and all biocides, except there was no correlation between the increase of MICs to thiomersal and MICs of antibiotics. It can be interpreted that thimerosal is not used frequently; it is used mainly as a preservative in a number of biological products that do not enable *Enterococci* to develop resistance against MLS antibiotics [72].

By screening the most abundant genes in the resistant isolates that are biocide tolerant, the efflux genes *mefA* and *mefE* were significantly increased than those in biocide with lower MICs. That indicates the possible roles of efflux in enhancing the resistance to both biocides and MLS antibiotics. Efflux pumps are major protective components of the bacterial cell wall that has been constitutively or inductively expressed and are responsible for the intrinsic and acquired resistance of many bacterial species to antimicrobials [73]. Bacterial active efflux compromises the effectiveness of antimicrobials and is crucial in cross-resistance to antibiotics and biocides [70,71,73,74]. In this direction, a fluorometric assay of the EtBr efflux has been used to quantify the efflux of selected highly resistant MLS isolates that showed higher MICs ≥ MIC_50_ or lower MICs < MIC_50_ to biocides. EtBr efflux has been assayed under limiting energy supply (absence of glucose and low temperature) and in the presence and absence of the approved efflux pump inhibitor verapamil [75]. Significantly, the MLS isolates with higher MICs to biocides >MIC_50_ extruded EtBr more than those with lower biocide MICs, indicating the essential role of efflux mechanism in cross-resistance to both antibiotics and biocides. It has been approved that there is a direct association between tolerance to biocides and antibiotic resistance since the mechanisms contributing to both are similar to changes in the cell permeability or the synthesis of efflux pumps [71,76].

## 4. Materials and Methods

### 4.1. Microorganisms

Nine hundred and thirteen clinical samples were collected from King Khalid Hospital, Ha’il, Saudi Arabia, from June 2019 to January 2020. Patient consent was obtained according to the hospital administration department’s routine hospital protocols in complete compliance with Helsinki declarations without any risk, burden, or danger to patients. The clinical specimens were collected from microbiological labs without direct patient contact.

### 4.2. Identification of Enterococcus *spp*.

The clinical specimens were cultivated on Bile esculin agar, Mannitol salt Agar, and MacConkey agar (Oxoid, Hampshire, UK) to isolate the *Enterococcus* spp. Further biochemical tests were performed to confirm the identification and to differentiate between *E. faecalis*, *E. faecium*, and other species of Group D *Enterococci* (Table 1) [26,27]. The biochemical tests were performed according to Elmer et al. [77].

### 4.3. Determination of Antibiotic Susceptibility and MICs of Isolates

All Enterococcal isolates were tested for their susceptibility to selected antibiotics using the disk diffusion method according to the Clinical and Laboratory Standards Institute (CLSI, 2015) [78,79]. The MICs of the tested antibiotics or biocides were determined by the agar dilution method according to CLSI, 2015. Furthermore, MIC_50_ and MIC_90_, the concentration that inhabited 50% or 90% of isolates, were calculated by determining the median, which corresponds to MIC_50,_ and 90th percental, which corresponds to MIC_90_ [80].

### 4.4. Determination of MLS Resistance Phenotypes by Triple Disk Diffusion Test

The test was performed according to Novotna et al. (2005) [43]. Standardized suspensions of the tested isolates (equivalent to the 0.5 McFarland) were prepared from overnight cultures in tryptone soya broth (TSB) and swabbed over the surface of Müeller-Hinton (MH) agar plates. Erythromycin (15 µg), clindamycin (2 µg), and lincomycin (2 µg) disks were placed in close proximity (20 mm) to each other over the agar surface. The plates were incubated for 16–18 h at 37 °C and then examined for the shape of inhibition zones if any. Significant ingrowth within zones up to the edges of each erythromycin, clindamycin, and lincomycin disk was considered constitutive resistance (cMLS) phenotype. Any flattening or blunting of the shape of the clindamycin zone indicates inducible resistance (iMLS) phenotype. Isolates resistant to erythromycin only but sensitive to clindamycin and lincomycin were considered to belong to M phenotypes. Resistance to lincomycin with sensitivity to clindamycin and erythromycin was considered an L phenotype.

### 4.5. PCR Detection of MLS Resistance Genes

PCR detection of MLS resistance encoding genes *ermA*, *ermC*, *ermB*, *msrA*, *mefA*, *mefE*, *ereA*, *lnuA*, and *mphC* genes was performed. The crude DNA was extracted using a Qiagen DNA extraction kit (Düsseldorf, Germany) [81] and stored at −80 °C [81,82]. The used primers are listed in Table 3.

### 4.6. Evaluation of the Efflux in MLS Resistant Isolates with Higher MIC to Biocide

In order to evaluate the efflux efficiency, a quantitative assay of ethidium bromide (EtBr) efflux was performed for selected isolates by fluorometric assay, according to Paixao et al. [73]. Twenty isolates that showed high MLS MIC > MIC_50_ to both antibiotics and biocides were selected to be compared with 20 isolates with lower MIC < MIC_50_ to both antibiotics and biocides.

The MICs of selected isolates for EtBr and the efflux pump inhibitor verapamil were determined by the broth microdilution method in 96-well microtiter plates according to the CLSI, 2015. Moreover, the MIC of EtBr in the presence of 1/5 of the MIC of verapamil was determined. In order to assure that the verapamil did not affect cellular viability, it was used at concentrations that did not exceed 1/5 of its MIC.

The selected isolates were grown in 10 mL of Luria-Broth (LB) broth to absorbance at 600 nm (OD600) of 0.6. The bacteria were then centrifuged at 14,000 rpm for 3 min. The pellet was washed twice with the same volume of PBS, and the OD600 of the cellular suspension was adjusted to 0.3. The accumulation assays were performed in 96-well fluorescence microtiter plates with a final volume of 100 µL. The conditions for the maximum accumulation (presence or absence of 0.4% glucose) of EtBr were first determined. Fifty µL of washed cell suspension was added to 50 µL of varying concentrations of EtBr in the absence or the presence of 0.4% glucose, and fluorescence was measured. ELISA reader 800 TS (BioTek, Winooski, VT, USA) was used to monitor the accumulation and extrusion of EtBr on a real-time basis. All the readings were made at excitation and emission wavelengths for EtBr (530 nm and 585 nm, respectively). All fluorescence data were acquired in cycles of 60 s, during a 1 h time interval, and at 25 °C. Each experiment was conducted in triplicate, and the results obtained were averaged.

After determining the optimum conditions for EtBr accumulation, the effect of verapamil on the accumulation of EtBr was determined. A volume of 50 µL of washed cell suspension was added to 50 µL PBS solutions containing EtBr (in sub-MIC) in the absence and the presence of 0.4% glucose and verapamil at concentrations that did not exceed 1/4 MIC. The fluorescence was measured as mentioned above, and the effect of verapamil on the fluorescence was determined. Each experiment was conducted in triplicate, and the results obtained were averaged.

The tested isolates were grown in 5 mL of LB, incubated at 37 °C for 18 h, centrifuged at 14,000 rpm for 5 min, and supernatants were discarded. The bacteria were loaded with EtBr (in sub-MIC) at 25 °C at 200 rpm for 1 h. Then, the pellets were washed with cold PBS and centrifuged at 13,000 rpm for 5 min. Supernatants were discarded, and each pellet was resuspended in 1 mL of cold PBS. A volume of 50 µL of each washed cell suspension was added in the 96-well microtiter plate containing (i) 50 µL of PBS without glucose, (ii) 50 µL PBS with 0.4% glucose, or (iii) 50µL PBS without glucose and with verapamil in concentrations that favor the maximum accumulation of EtBr. Aliquots of 100 µL were assayed at 37 °C with continuous fluorescence measurement as described previously, and each experiment was performed in triplicate. The efflux of EtBr is expressed in terms of relative fluorescence (RF), which is obtained from the comparison between the fluorescence observed for the bacteria in the presence or absence of glucose and the negative efflux control in the absence of glucose and the presence of verapamil following the formula

$$\mathrm{RF}=\frac{\mathrm{Measured}\;\mathrm{fluorescence}\;\mathrm{in}\;\left(\mathrm{PBS}-\mathrm{glucose}\right)—\mathrm{Measured}\;\mathrm{fluorescence}\;\mathrm{in}\;\left(\mathrm{PBS}+\mathrm{glucose}\right)}{\mathrm{Measured}\;\mathrm{fluorescence}\;\mathrm{in}\;\left(\mathrm{PBS}\;-\mathrm{glucose}+\mathrm{verapamil}\right)}$$

## 5. Conclusions

This study aimed to characterize the resistance to MLS antibiotics phenotypically and genotypically. The target-site modification of bacterial 50S ribosomal subunit was the most prevalent mechanism of resistance to MLS antibiotics. The constitutive resistance to MLS was the most predominant phenotype. In consistence with this, rRNA methylase *erm* genes *ermB*, *A*, and *C* were highly distributed among *Enterococci* isolates. The MLS-inactivating enzymes encoding genes were detected in the tested isolates, particularly esterase encoded by the *ereA* gene. On the other hand, the *lnuA* gene, which is mainly associated with lincomycin resistance, was the least detected. While the least resistance of tested isolates was detected against clindamycin, the higher rates were detected against erythromycin, azithromycin, and clarithromycin, represented as MLS or M phenotypes. There was a significant correlation between the reduced susceptibility of isolates to the commonly used biocides and the resistance to MLS antibiotics. Importantly, the increased efflux was observed phenotypically, and its encoding genes in the resistant MLS isolates showed reduced susceptibility to biocides. That could indicate the increased role of efflux in conferring resistance to both antibiotics and biocides.

## Figures and Tables

**Table 1:** Biochemical characterization difference between *E. faecalis* and *E. faecium*.

Test	*E. faecalis*	*E. faecium*
Catalase	−	−
Oxidase	−	−
Motility	Non-motile	Non-motile
Growth in 6.5% NaCl	+	+
Growth at 45 °C	+	+
Lactose fermentation	+	+
Mannitol fermentation	+	+
Growth in 0.04% tellurite	+	−
Arabinose fermentation	−	+

**Table 2:** MLS phenotypes.

Isolates	Resistance Phenotype				Total
	cMLS	iMLS	M	L	
*E. faecalis*	42 (76.4%)	2 (3.6%)	10 (18.2%)	1 (1.8%)	55
*E. faecium*	34 (79.1%)	1 (2.3%)	7 (16.3%)	1 (2.3%)	43
Other *Enterococci*	7 (70%)	1 (10%)	2 (20%)	0	10
Total	83 (76.8%)	4 (3.7%)	19 (17.6%)	2 (1.9%)	108

cMLS = Constitutive macrolides, lincosamides, and streptogramin B resistance phenotype. iMLS = Inducible macrolide, lincosamide, and streptogramin resistance phenotype. M = Macrolides and streptogramin B or macrolides resistance phenotype. L = Lincosamides inactivation resistance phenotype.

**Table 3:** Primers used in PCR for detection of resistance genes.

Gene	Primer	Primer Sequence (5′-3′)	References
*erm*A	F	AAGCGGTAAACCCCTCTGA	[83]
	R	TTCGCAAATCCCTTCTCAAC	
*erm*B	F	CTATCTGATTGTTGAAGAAGGATT	[49]
	R	GTTTACTCTTGGTTTAGGATGAAA	
*erm*C	F	AATCGTCAATTCCTGCATGT	[83]
	R	TAATCGTGGAATACGGGTTTG	
*msr*A	F	TCCAATCATTGCACAAAATC	[49]
	R	AATTCCCTCTATTTGGTGGT	
*mef*A	F	CGTAGCATTGGAACAGC	[84]
	R	TGCCGTAGTACAGCCAT	
*mef*E	F	CGTAGCATTGGAACAGC	[84]
	R	TCGAAGCCCCCTAATCTT	
*ere*A	F	AACACCCTGAACCCAAGGGACG	[85]
	R	CTTCACATCCGGATTCGCTCGA	
*lnu*A	F	GGTGGCTGGGGGGTAGATGTATTAACTGG	[68]
	R	GCTTCTTTTGAAATACATGGTATTTTTCGATC	
*mph*C	F	ATGACTCGACATAATGAAAT	[86]
	R	CTACTCTTTCATACCTAACTC	

## Data Availability

Not applicable.

## Supplementary Materials

The following supporting information can be downloaded at: https://www.mdpi.com/article/10.3390/antibiotics12030461/s1, Table S1: MLS susceptibility patterns with number and percentage of isolates; Table S2: The distribution MLS resistance genes among the resistant phenotypes with numbers and percentages; Table S3: MIC range, MIC_50_, and MIC90 to MLS antibiotics; Table S4: MIC range, MIC_50_, and MIC90 to biocides.
