# Supplementary material for: Clinical Resistant Strains of Enterococci and Their Correlation to Reduced Susceptibility to Biocides: Phenotypic and Genotypic Analysis of Macrolides, Lincosamides, and Streptogramins
Source: Antibiotics (Basel). 2023 Feb 24;12(3):461. doi: 10.3390/antibiotics12030461 (PMC10044631; doi:10.3390/antibiotics12030461)
Supplement: Supplementary file 1 [file antibiotics-12-00461-s001.zip › antibiotics-2216900-supplementary.pdf]

Supplementary Data

Table S1. MLS susceptibility patterns with number and percentage of isolates

Pattern	Resistance to antibiotics							*E. faecalis*		*E. faecium*		*Other Enterococci spp*		Total	
	E	AZM	CLR	SP	L	DA	QD	No	%	No	%	No	%	No	%
I								12	17.9	9	17.3	2	16.7	23	17.6
II	R	R	R	R	R	R	R	24	35.8	16	30.8	4	33.3	44	33.6
III	R	R	R	R				5	7.5	3	5.8	1	8.3	9	6.9
IV					R	R	R	1	1.5	1	1.9			2	1.5
V	R							3	4.5	2	3.8			5	3.8
VI	R	R		R	R	R	R	1	1.5	1	1.9			2	1.5
VII	R	R	R		R	R	R	1	1.5	1	1.9	1	8.3	3	2.3
VIII	R	R			R			2	3.0	1	1.9			3	2.3
IX	R				R			3	4.5	2	3.8	1	8.3	6	4.6
X	R			R	R	R	R	1	1.5	1	1.9			2	1.5
XI	R	R	R	R	R		R	5	7.5	7	13.5	1	8.3	13	9.9
XII	R			R	R			1	1.5	1	1.9			2	1.5
XIII					R			3	4.5	3	5.8	2	16.7	8	6.1
XIV	R				R	R		3	4.5	2	3.8			5	3.8
XV			R		R			1	1.5	1	1.9			2	1.5
XVI	R	R	R		R			1	1.5	1	1.9			2	1.5

***R= Resistant; Antibiotics: E=** Erythromycin**, AZM= Azithromycin, CLR= Clarithromycin, SP= Spiramycin, L= Lincomycin, DA= Clindamycin**, and **QD= Quinupristin/Dalfopristin.**

Table S2. The distribution MLS resistance genes among the resistant phenotypes with numbers and percentages

Pattern	Resistance gene profile												Resistance phenotype	Total No. (%)
	ermA	*ermB*	*ermC*	*msrA*	*mefA*	*mefE*	*ereA*	*lnuA*	*mphC*	*E. faecalis*	*E. faecium*	Other *Enterococci*		
I	+	+	+	-	-	-	-	-	-	3	2		cMLS	5 (4.6%)
I$_1$	+	+	+	-	-	-	+	-	-	1			cMLS	1 (0.9%)
II	+	+	-	-	-	-	-	-	-	2			cMLS	2 (1.9%)
II$_1$	+	+	-	-	+	+	+	-	-	13	11	3	cMLS	27 (25%)
II$_2$	+	+	-	-	-	-	-	+	-	1			cMLS	1 (0.9%)
II$_3$	+	+	-	-	-	-	-	-	+	2	1	1	cMLS	4 (3.7%)
II$_4$	+	+	-	-	-	-	+	-	+	1	5	1	cMLS	7 (6.5%)
III	-	+	+	-	-	-	+	-	-	1	1		iMLS	2 (1.9%)
II-a	+	+	-	-	-	-	-	-	-	1		1	iMLS	2 (1.9%)
V	-	+	-	-	-	-	-	-	-	5	5	1	cMLS	11 (10.2%)
V$_1$	-	+	-	+	-	-	-	-	-	1	1		M	2 (1.9%)
V$_{11}$	-	+	-	+	-	+	-	-	-	1	1		M	2 (1.9%)
V$_{12}$	-	+	-	+	+	-	-	-	-	1			M	1 (0.9%)
V$_{13}$	-	+	-	+	+	+	-	-	-	1	1		M	2 (1.9%)
V$_{14}$	-	+	-	+	+	+	-	-	+	1	1		M	2 (1.9%)
V$_2$	-	+	-	-	+	+	+	-	-	12	9	1	cMLS	22 (20.4%)
V$_{21}$	-	+	-	-	-	-	+	-	+	1	1		cMLS	2 (1.9%)
V$_3$	-	+	-	-	+	+	+	-	-	1			cMLS	1 (0.9%)
V$_{31}$	-	+	-	-	+	+	-	-	-	5	3	1	M	9 (8.3%)
VI$_1$	-	-	-	-	-	-	+	-	-		1		L	1 (0.9%)
VI$_2$	-	-	-	-	-	-	+	+	-	1			L	1 (0.9%)
VII	-	-	-	-	+	+	-	-	-			1	M	1 (0.9%)

Table S3. MIC range, MIC_{50}, and MIC_{90} to MLS antibiotics

Antibiotic	*E. faecalis*			*E. faecium*			Other *Enterococci* spp		
	MIC range (µg/ml)	MIC_{50}	MIC_{90}	MIC range (µg/ml)	MIC_{50}	MIC_{90}	MIC range (µg/ml)	MIC_{50}	MIC_{90}
Erythromycin	0.125 - 1024	8	614.4.	0.125 - 1024	8	870.4	0.125 - 1024	6	435.2
Azithromycin	0.125 - 1024	8	512	0.125 - 1024	12	512	0.125 - 512	4	396.8
Clarithromycin	0.125 - 1024	1	614.4	0.125 - 1024	0.5	437.9	0.125 - 512	0.75	217.6
Spiramycin	0.125 - 1024	1	512	0.125 - 1024	0.5	512	0.125 - 265	0.375	217.6
Lincomycin	0.125 - 1024	16	512	0.125 - 1024	16	870.4	0.125 - 512	12	435.2
Clindamycin	0.125 - 1024	8	614.4.	0.125 - 1024	8	512	0.125 - 1024	6	870.4
Quinupristin/ Dalfopristin	0.125 - 1024	4	314.4.	0.125 - 1024	4	256	0.125 - 256	3	256

Table S4. MIC range, MIC_{50}, and MIC_{90} to biocides

Biocides	*E. faecalis*			*E. faecium*			Other *Enterococci* spp		
	MIC range (µg/ml)	MIC_{50}	MIC_{90}	MIC range (µg/ml)	MIC_{50}	MIC_{90}	MIC range (µg/ml)	MIC_{50}	MIC_{90}
Triclosan	0.4 - 7.0	2.0	5.5	0.1 - 6.0	2.0	4.8	0.5 – 6.0	2.2	5.4
Cetrimide	0.6 – 3.0	1.4	2.68	0.5 - 10.0	3.4	5.7	0.5 – 5.0	1.45	3.6
Glutaraldehyde	0.5 - 1.8	0.75	1.45	0.1 - 0.9	0.65	0.88	0.2 - 0.9	0.43	0.8
Thiomersal	0.2 - 3	0.7	2.6	0.8 - 6.4	1.8	5	0.5 - 3	1.3	2.6
Chlorocresol	200 - 600	280	560	150 - 500	260	480	150 - 600	280	570
Povidone-iodine	4200-5600	5000	5500	4100-5600	4800	5300	900-1500	1050	1400
